# Caspase-1-mediated extracellular vesicles derived from pyroptotic alveolar macrophages promote inflammation in acute lung injury

**DOI:** 10.7150/ijbs.66477

**Published:** 2022-01-24

**Authors:** Xichun Qin, Yueyuan Zhou, Caili Jia, Zhixiang Chao, Hao Qin, Jingtian Liang, Xiucheng Liu, Zhiwei Liu, Teng Sun, Yanliang Yuan, Hao Zhang

**Affiliations:** 1 Thoracic Surgery Laboratory, The First College of Clinical Medicine, Xuzhou Medical University, Xuzhou 221006, Jiangsu Province, China.; 2 Department of Thoracic Surgery, Affiliated Hospital of Xuzhou Medical University, 99 West Huaihai Road, Xuzhou 221006, Jiangsu, China.; 3 Department of Thoracic and Cardiovascular Surgery, the Affiliated Drum Tower Hospital of Nanjing University Medical School, Nanjing 210008, China.; 4 Department of Clinical Medical Engineering, The First Affiliated Hospital of Nanjing Medical University, Nanjing 210029, China.; 5 Public Experimental Research Center, Xuzhou Medical University, Xuzhou 221006, Jiangsu Province, China.

**Keywords:** extracellular vesicles, pyroptosis, acute lung injury, alveolar macrophage, caspase-1

## Abstract

The occurrence and development of acute lung injury (ALI) involve a variety of pathological factors and complex mechanisms. How pulmonary cells communicate with each other and subsequently trigger an inflammatory cascade remains elusive. Extracellular vesicles (EVs) are a critical class of membrane-bound structures that have been widely investigated for their roles in pathophysiological processes, especially in immune responses and tumor progression. Most of the current knowledge of the functions of EVs is related to functions derived from viable cells (e.g., microvesicles and exosomes) or apoptotic cells (e.g., apoptotic bodies); however, there is limited understanding of the rapidly progressing inflammatory response in ALI. Herein, a comprehensive analysis of micron-sized EVs revealed a mass production of 1-5 μm pyroptotic bodies (PyrBDs) release in the early phase of ALI induced by lipopolysaccharide (LPS). Alveolar macrophages were the main source of PyrBDs in the early phase of ALI, and the formation and release of PyrBDs were dependent on caspase-1. Furthermore, PyrBDs promoted the activation of epithelial cells, induced vascular leakage and recruited neutrophils through delivery of damage-associated molecular patterns (DAMPs). Collectively, these findings suggest that PyrBDs are mainly released by macrophages in a caspase-1-dependent manner and serve as mediators of LPS-induced ALI.

## Introduction

Acute lung injury (ALI) is non-cardiogenic acute progressive hypoxic respiratory failure that is caused by a variety of intrapulmonary or exogenous factors. Its characteristic pathological changes include pulmonary edema, exudation, and massive accumulation of inflammatory cells caused by increased pulmonary microvascular permeability^1^, ^2^. The uncontrolled inflammatory response causes the destruction of epithelial and endothelial barriers, which ultimately leads to the high mortality rate in acute respiratory distress syndrome (ARDS) 3. Globally, ARDS patients account for about 10% of intensive care unit hospitalized patients, and most patients experience deterioration within 2-5 days after hospitalization 4. Therefore, it is urgently needed to clarify the molecular mechanism of pulmonary inflammation to interrupt the progression of ALI to ARDS.

Evidence is accumulating that extracellular vesicles (EVs) play a vital role in cell-to-cell crosstalk and that they may be involved in inflammation, immune response, blood coagulation, tumorigenesis, and host-microbe interactions 5. EVs are a broad term, and they include exosomes, microvesicles, apoptotic bodies, retrovirus-like particles 6 and oncosomes 7. Research studies on EVs have challenged the currently accepted paradigm of inflammatory disease mechanisms. EV-mediated transfer of signals among pulmonary cells is also now recognized as a novel mechanism of the development of lung inflammation and injury 8. The types of EVs detected or isolated from bronchoalveolar lavage fluid (BALF), including exosomes 9, microvesicles 10, or apoptotic bodies 11, depend on the type of harmful stimulus and severity of disease. However, most of the research on EVs tends to focus on EVs below 1000 nm, such as exosomes or microvesicles, and little is known about the functions of other larger EVs. Although the understanding of the inflammatory response mechanism mediated by exosomes and microvesicles has improved, there are still limited data on how EVs mediate the acute out-of-control inflammatory response mechanism of the lung.

Pyroptosis is a new type of programmed cell death, which has been recently discovered and confirmed 12, 13. The purulent cascade caused by gram-negative bacterial infections leads to the release of a large amount of endotoxin (LPS), which mediates the activation of a variety of caspases, including caspase-1; it also causes multiple gasdermin family members, including Gasdermin D (GSDMD), to undergo shearing and multimerization, resulting in cell perforation. Compared with apoptosis, pyroptosis occurs more rapidly and is accompanied by the release of a large number of proinflammatory factors. Pyroptotic cells can also amplify the inflammatory response by releasing apoptosis-associated speck-like protein specks (ASC specks) that are rich in inflammasome signals, which are phagocytosed by neighboring cells 14. Pyroptosis plays a key role in the development of ALI inflammation^15^, ^16^. Although some studies have found that a certain number of exosomes and microvesicles are derived during pyroptosis 17-19, systematic studies on pyroptosis-dependent extracellular vesicles are limited. EVs derived from pyroptotic cells (denoted as PyrEVs) are also not well defined. In particular, plasma membrane blistering similar to apoptosis occurs in the process of pyroptosis, leading to the formation of micron-sized vesicles (1-5 μm) called pyroptotic bodies (denoted as PyrBDs) 20, 21. To date, EVs derived from pyroptotic cells (PyrEVs) have not been systematically defined; moreover, due to the large size of PyrBDs, conventional EV separation and purification processes usually remove such larger EVs from the sample. Their existence is only supported by morphological evidence from in vitro experiments. However, it is still unknown whether PyrEVs are produced in inflammation-related diseases, such as ALI, and their biological role remains uncertain.

In this study, we conducted detailed characterization of 1-5 μm micro-sized EVs (MsEVs) in BALF in the early phase of ALI and studied their biological activities. We confirmed that alveolar macrophages released caspase-1-mediated pyroptosis-dependent micron-sized vesicles in the early phase of LPS-induced ALI in mice. Those vesicles encapsulated with a variety of damage-associated molecular patterns (DAMPs) mediated pulmonary epithelial cell activation, caused vascular interstitial edema, and participated in the recruitment of neutrophils. Furthermore, we characterized the presence of cleaved caspase-1 and its substrate protein in PyrBDs. These results helped us to understand the relevant characteristics of PyrEVs and their inflammatory effects in ALI.

## Results

### MsEVs are released in the early phase of the LPS-induced ALI mouse model

**Figure 1:**
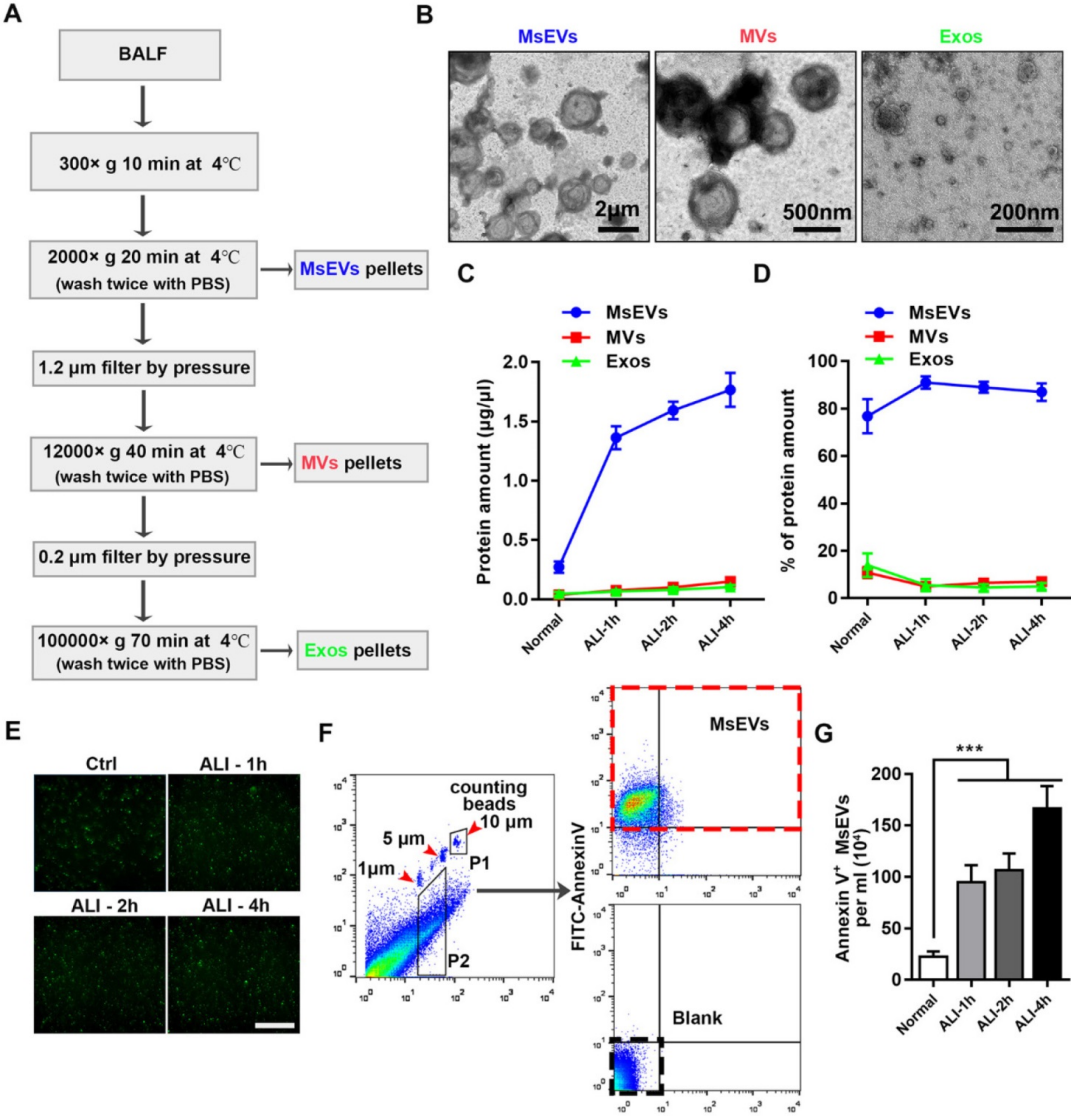
Mass release of micrometer-sized extracellular vesicles in the early phase of ALI C57BL/6J mice induced by LPS. (A) Protocol to harvest micron-sized vesicles, microvesicles, and exosomes. BALF was harvested from mice instilled or not instilled with LPS, and extracellular vesicles were separated by serial centrifugation. The pellets were washed with PBS and then centrifuged twice to remove soluble factors or impurities outside the vesicles. (B) Representative transmission electron micrograph of extracellular vesicles pellets derived from differential centrifugation. Bar (from left to right) = 2 μm, 500 nm, 200 nm. (C) Protein concentration of three types of EVs and (D) the percentages of each type of EVs. (E) Immunostaining showing positive expression of FITC-Annexin V in the pellets. The pellets were incubated with FITC-labeled Annexin V^+^ for 15 min, washed twice with PBS, and observed under a fluorescence microscope. (F) The number of MsEVs was determined by flow cytometry. One- and 5-μm-diameter beads and 10-μm counting beads were used to gate 1-5 μm-sized EVs. Annexin V^+^ vesicles were counted as MsEVs (P2 * Annexin V^+^ rate / P1 * number of counting beads). Data are expressed as mean ± SD, n = 6. ***P < 0.001.

To gain insights into the kinetics of EV release, BALF samples from an ALI mouse model instilled with LPS at different time points (1 h, 2 h, and 4 h) and a normal group (without tracheal drip of LPS) were collected; MsEVs, microvesicles, and exosomes were obtained by differential centrifugation (protocol, Figure 1A). The isolated EVs were heterogenous in size, as shown in transmission electron microscopy images (Figure 1B). Protein quantification results showed that MsEVs had the highest protein ratio, especially during ALI, occupying about 90% of total protein (Figure 1C and D). This suggested that MsEVs might play an important role in the ALI process.

Before investigating whether these MsEVs are rich in PyrBDs, we first quantified these MsEVs. Pyroptotic cells displayed swelling and produced multiple bubble-like protrusions before rupture of the plasma membrane. The bubble-like protrusions in pyroptotic cells then progressed into 1-5 μm EVs (PyrBDs) 20, 21. Compared with traditionally studied EVs, there is still a lack of definition of PyrEVs; the basic standard for characterizing PyrEVs, especially the basic characteristics of PyrBDs, has not been established in this field. Thus, we first defined the MsEVs separated from BALF as those with (i) sizes between 1 μm and 5 μm, and (ii) positive expression of plasma membrane phospholipid-binding protein. It is worth noting that except for the well-defined size of exosomes and microvesicles, the definition of apoptotic bodies and other vesicles remains vague. Our definition was not strictly exclusive but helped us to distinguish the PyrBD group in terms of size. The isolated EVs were then immunostained with FITC-Annexin V. Fluorescence microscopy revealed that Annexin V was expressed in the EVs (Figure 1E). EVs between 1 μm and 5 μm in diameter were gated using calibration beads and quantified using counting beads by flow cytometry (Figure 1F). The number of MsEVs was calculated by the number of counting beads multiplied by the ratio of FITC-Annexin V antibody-positive events to counting bead events in flow cytometry plots 22. Figure 1G shows that high levels of MsEVs were released by ALI 1 h compared with the normal group, and the number of MsEVs remained at a high level at time points 2 h and 4 h. Together, these findings demonstrated that a large number of MsEVs were released in LPS-induced ALI.

### Alveolar macrophages are the main contributors to MsEVs in the early phase of ALI

**Figure 2:**
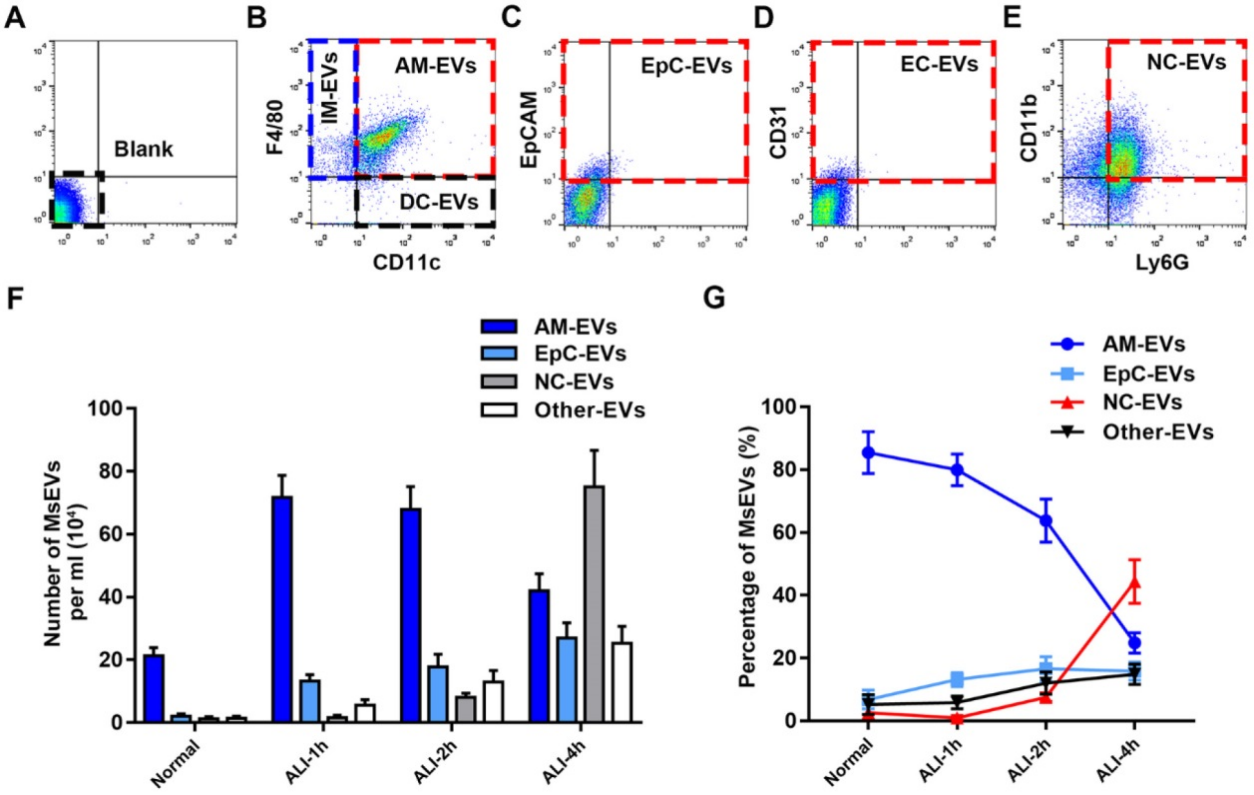
Characterization of MsEVs from different cell types. (A-E) The origin of BALF MsEVs classified by flow cytometry. CD11c^+^ and F4/80^+^ events designate alveolar macrophages; EpCAM^+^—epithelial cells; other events (such as CD31^+^ for endothelial cells, CD11c^+^ F4/80^-^ for dendritic cells, CD11c^-^ F4/80^+^ for interstitial macrophages) were pooled into the Other group. (F and G) Quantitative measurement of MsEVs. MsEVs from different cell sources were detected at different time points in the early phase of the ALI model (n=6). Data are expressed as the mean ± SD. AM, alveolar macrophage; IM, interstitial macrophage; DC, dendritic cell; EpC, epithelial cell; NC, neutrophil; EC, endothelial cell.

The progress of LPS-induced ALI at the early stage was investigated after determining mass production of MsEVs. To reliably identify MsEVs by flow cytometry in BALF, we used specific precursor cell markers to analyze the cell source of MsEVs 9, 10. We determined the dynamics of MsEVs release from alveolar macrophages, epithelial cells, neutrophils, endothelial cells, dendritic cells, and interstitial macrophages within the alveolar space during the early phase of LPS-induced ALI in mice (Figure 2A-E). Following LPS induction, the number of MsEVs in BALF rapidly increased. MsEVs originating from alveolar macrophages were apparently the predominant MsEV population at 1 h after LPS induction (Figure 2B) and decreased thereafter (Figure 2F and G). Epithelial cell-derived MsEVs continued to increase from the time of LPS challenge (Figure 2C, F, and G). Additionally, MsEVs derived from other cells were detected in small proportion after LPS induction (Figure 2G). Few neutrophil-derived MsEVs were detected at 1-2 h after LPS, but they subsequently increased at 4 h (Figure 2E and F). Consistently, it was observed that neutrophils postponed their involvement in the inflammatory response via morphological alterations detected by hematoxylin-eosin (HE) staining (Supplemental Figure 1A) and results of neutrophil counting and myeloperoxidase (MPO) activity (Supplemental Figure 1C and D). Compared with the normal group, interstitial edema developed in ALI 1 h, and the alveolar wall thickened with pulmonary vascular leakage as indicated by Evans blue dye bound tightly to albumin (EBA) extravasation (Supplemental Figure 1A and B). TNF-α, IL-1β, and IL-6 are common biomarkers of inflammatory damage in lung tissues 23, 24. The expression of TNF-α, IL-6, and IL-1β at ALI groups was upregulated compared with the normal group, and interestingly, they peaked at ALI 1 h (Supplemental Figure 1E). Notably, an influx of neutrophils into the interstitium was observed until ALI 4 h (Supplemental Figure 1A and C). These findings reflected the delayed involvement of neutrophils in the inflammatory response; hence, neutrophils may not mediate the communication of lung cell inflammatory response in the early phase of ALI. According to these results, we identified the important role of alveolar macrophages as the source of MsEVs in the early phase of ALI; thus, we focused on the MsEVs of the normal group and the ALI-1h group, where the main source of MsEVs were alveolar macrophages, and MsEVs derived from alveolar macrophages in the ALI-1h group had an absolute quantitative advantage.

### MsEVs derived from alveolar macrophages mediate inflammation in the early phase of ALI

**Figure 3:**
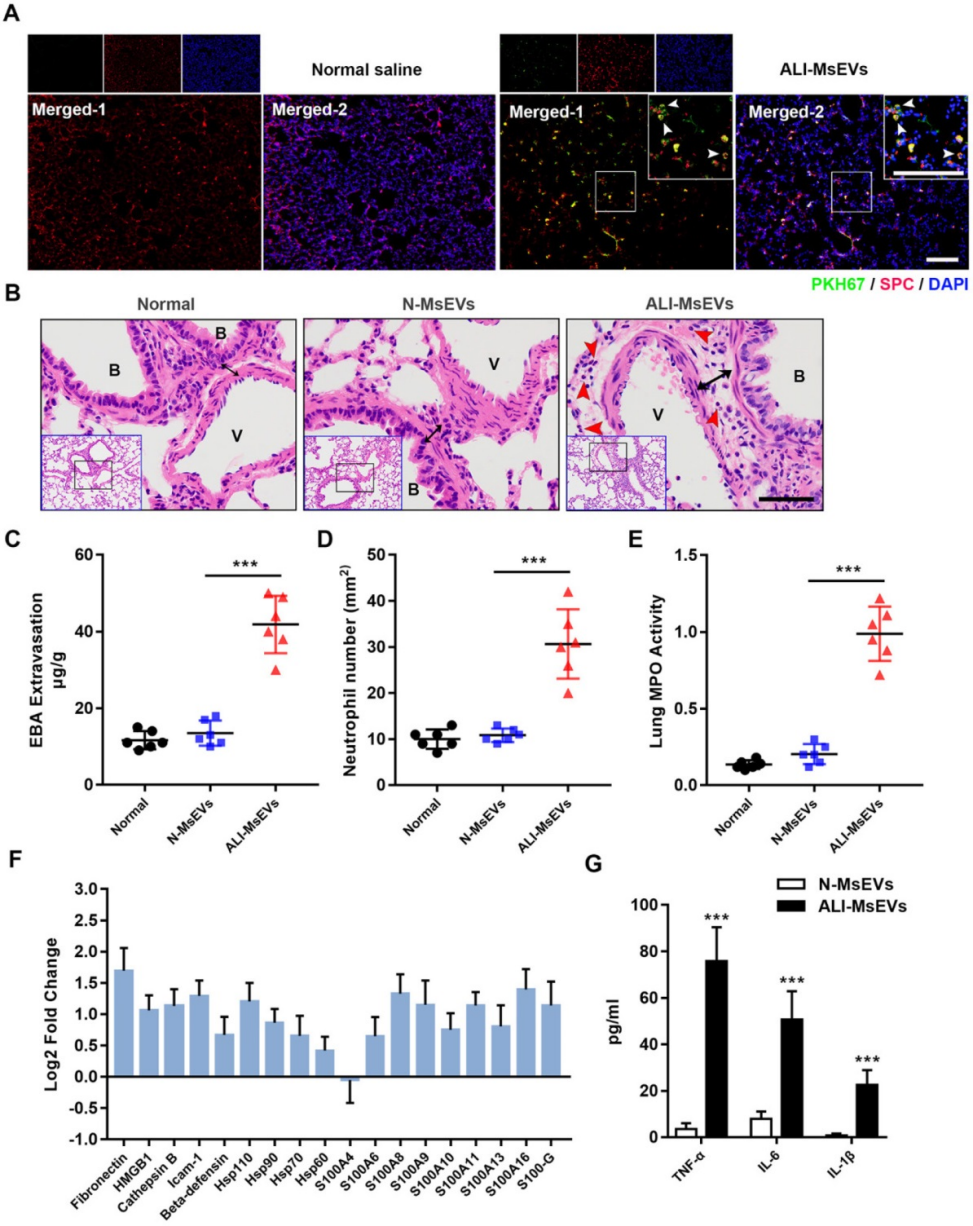
MsEVs derived from alveolar macrophages in the early stage of ALI encapsulate DAMPs and mediate inflammation. (A) After tracheal drip of PKH67-labeled PyrBDs for 4 h, immunofluorescent staining showed that PKH67 co-localized with SPC, indicating the uptake of PyrBDs by lung epithelial cells. The white arrow points to undegraded PyrBDs (green); bar = 100 μm. The number of EVs is one million per mouse. SPC (red); DAPI (blue). (B) H&E-stained cross-section of the lung from N-MsEVs (MsEVs from the normal group) and ALI-MsEVs (MsEVs from the ALI-1h group) exposed mice at 4 h shows interstitial edema (indicated by black arrows). The red arrow points to neutrophils. Scale bars: 50 μm. B, trachea; V, blood vessel. Images are representative of six animals. (C) Assessment of the degree of lung vascular leakage (EBA extravasation). (D) Quantitative analysis of neutrophil infiltration in the lungs. (E) Quantitative analysis of lung tissue MPO activity. (F) PRM for whole-protein analysis of MsEVs, from which DAMP-related molecular proteins were screened. EVs pellets were isolated from BALF collected from control mouse or 1 h post-LPS. The results are expressed as log2 fold change (n = 5). (G) TNF-α, IL-6, and IL-1β content of MsEVs. The EVs pellets were ultrasonically broken, the supernatant was obtained by centrifugation at 14,000*g*, and cytokine content was detected by ELISA kit. Compared with N-MsEVs, ALI-MsEVs contain high levels of TNF-α, IL-6, and IL-1β. Data are expressed as the mean ± SD, n = 6. ***P < 0.001.

To explore the biological effects of MsEVs derived from alveolar macrophages, we instilled MsEVs through the trachea to achieve intrapulmonary delivery of MsEVs. First, PKH-67-labeled MsEVs collected from the ALI-1h group were used to observe their uptake by the lung tissue by immunofluorescence. After 4 h of instillation, the results of immunofluorescence observation of the lung tissue showed that there were many specific green fluorescence signals on the whole cross-section of the lung (Figure 3A). SPC was used to mark type II alveolar epithelial cells 25. We observed co-localization of PKH-67 signals and SPC in lung cross sections, which further confirmed the uptake of PyrBDs by type 2 alveolar epithelial cells (Figure 3A). Next, we performed pathological examinations of the lung tissue. The results showed that, compared with the MsEVs from the normal group, the MsEVs from the ALI-1h group caused interstitial edema and neutrophil infiltration in the lung tissue of mice (Figure 3B-E). That suggested that MsEVs derived from alveolar macrophages during ALI could mediate the inflammatory response.

Through whole-protein analysis of MsEVs, we screened 18 types of danger-associated molecular pattern proteins (DAMPs), including fibronectin, HMGB1, heat-shock protein, and s100s, which have been reported to play significant proinflammatory and pro-damage effects in ALI 26. Quantitative analysis revealed that the expression of DAMPs was generally higher in MsEVs derived from the ALI-1h group than in those from the normal group (Figure 3F). The concentration of inflammatory factors was quantified by ELISA because it was difficult to accurately detect them by the PRM technology. We mainly checked the expression of TNF-α, IL-6, and IL-1β in MsEVs. The results showed that the expression of inflammatory cytokines was much higher in MsEVs derived from the ALI-1h group than in those from the normal group (Figure 3G). The above results suggested that MsEVs derived from alveolar macrophages during ALI might mediate the inflammatory response by transferring DAMPs.

### Requirement of caspase-1 in the release of MsEVs and inflammatory injury

**Figure 4:**
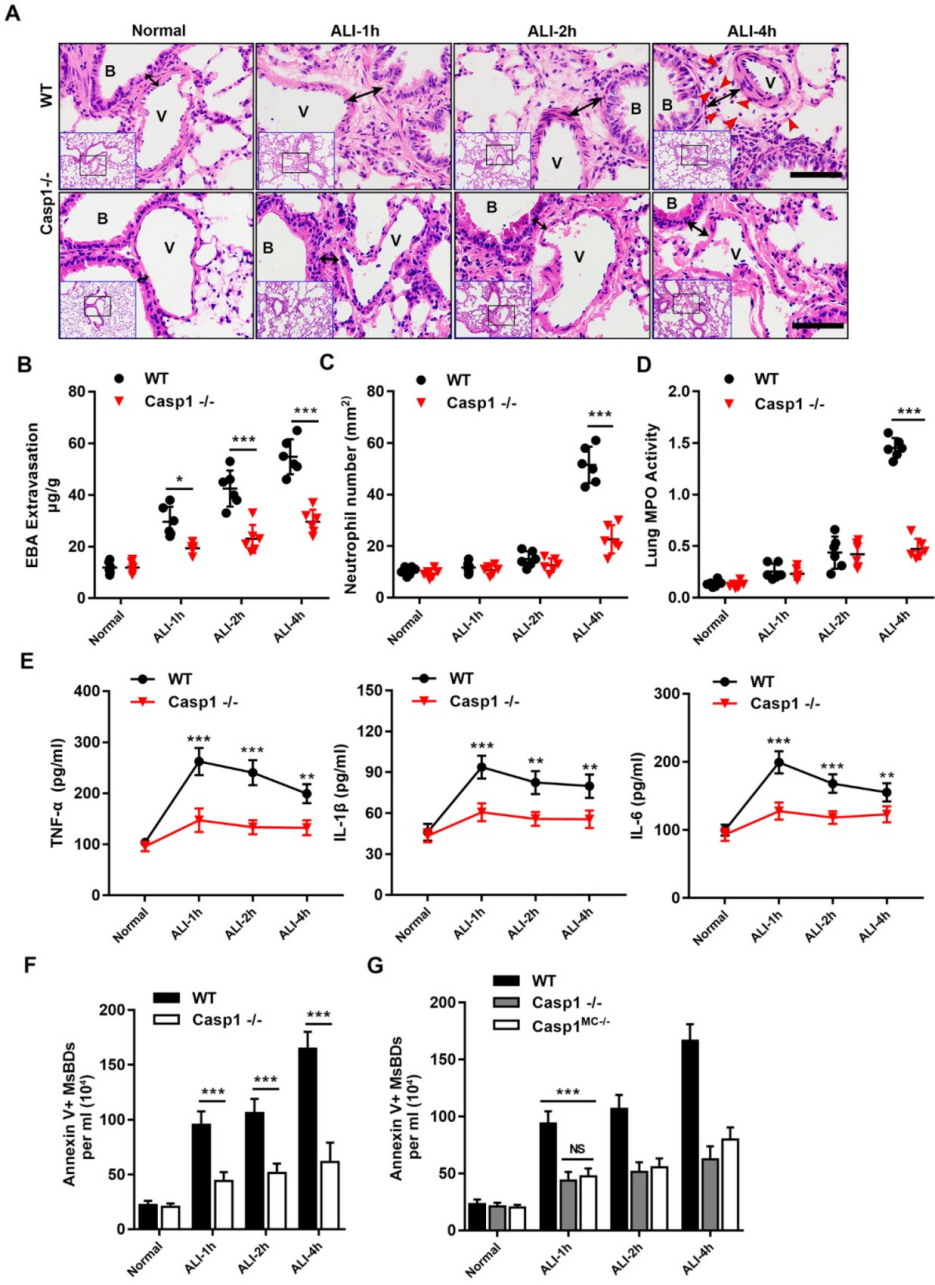
Requirement for caspase-1 in mice in mediating inflammatory damage and release of MsEVs. (A) H&E-stained cross-section of the lung from LPS-exposed WT and Casp1^-/-^ mice at 1, 2, and 4 h shows interstitial edema (indicated by black arrows) in the ALI groups. The red arrow points to neutrophils. Scale bars: 50 μm. B, trachea; V, blood vessel. Images are representative of six animals. Quantitative analysis of (B) lung vascular leakage (EBA extravasation), (C) neutrophil infiltration and (D) lung tissue MPO activity. Lack of caspase-1 significantly reduced LPS-induced vascular leakage and neutrophil infiltration. (E) Quantification of cytokines. TNF-α, IL-1β, and IL-6 expression increased in the WT groups compared with the Casp1^-/-^ groups (n=6). Data are expressed as the mean ± SD. **P < 0.01; ***P < 0.001. (F) The number of MsEVs was determined by flow cytometry. The same dose of LPS was instilled into the trachea; compared with the WT ALI model group, the amount of MsEVs significantly decreased in Casp1^-/-^ mice. (G) Specifically knocking out macrophage caspase-1 in mice, the release of MsEVs triggered by LPS was significantly reduced. Data are expressed as the mean ± SD. NS, no significant difference; **P < 0.01; ***P < 0.001.

The morphological evidence of PyrBDs is based on the classical pyroptosis model, in which the activation of caspase-1 is critical to induce pyroptosis 20. To determine whether the release of MsEVs is dependent on pyroptosis, a caspase1-knockout mouse model was established followed by LPS challenge. HE staining of lung tissues after disrupting caspase-1 expression showed a significant attenuation of lung damage, and vascular interstitial edema was markedly reduced. Additionally, the number of MsEVs significantly decreased in the Casp1^-/-^ group (Figure 4B), suggesting that the release of MsEVs in pyroptosis was dependent on caspase-1. Furthermore, neutrophil infiltration in the lung interstitium that was marked by EBA extravasation was hardly observed at 4 h after LPS induction (Figure 4A). Consistently, lung vascular leakage marked by EBA extravasation, neutrophil infiltration, and lung tissue MPO activity was significantly downregulated in the Casp1^-/-^ group compared with the wild type (WT) (Figure 4C-E). The expression levels of inflammatory cytokines TNF-α, IL-6, and IL-1β were also lower in the Casp1^-/-^ group compared with the WT group (Figure 4F). Collectively, these results indicated that the lack of caspase-1 hindered neutrophil infiltration, production of inflammatory cytokines, and release of MsEVs. To verify that the release of MsEVs derived from alveolar macrophages was dependent on caspase-1 in vivo, a caspase-1 macrophage-specific deletion mouse model (Casp1^MC-/-^) was established by crossing Casp1^f1^°^x/f1^°^x^ (carrying a LoxP site-flanked Casp1 for tissue-specific deletion of caspase-1) with Lyz2-cre mice. The quantity of MsEVs isolated from BALF of the Casp1^MC-/-^ mice as well as that of the Casp1^-/-^ group was significantly downregulated compared with the WT group (Figure 4G). It is worth noting that pathological examination of lung tissue of Casp1^MC-/-^ mice showed its significant resistance to LPS (Supplemental Figure 2A-E). These results clearly indicated that caspase-1 mediated the secretion of MsEVs by alveolar macrophages in vivo and that the samples of MsEVs isolated from BALF of ALI mouse might be rich in PyrBDs.

### Activation of epithelial cells by PyrBDs via the p38 MAPK signaling pathway

**Figure 5:**
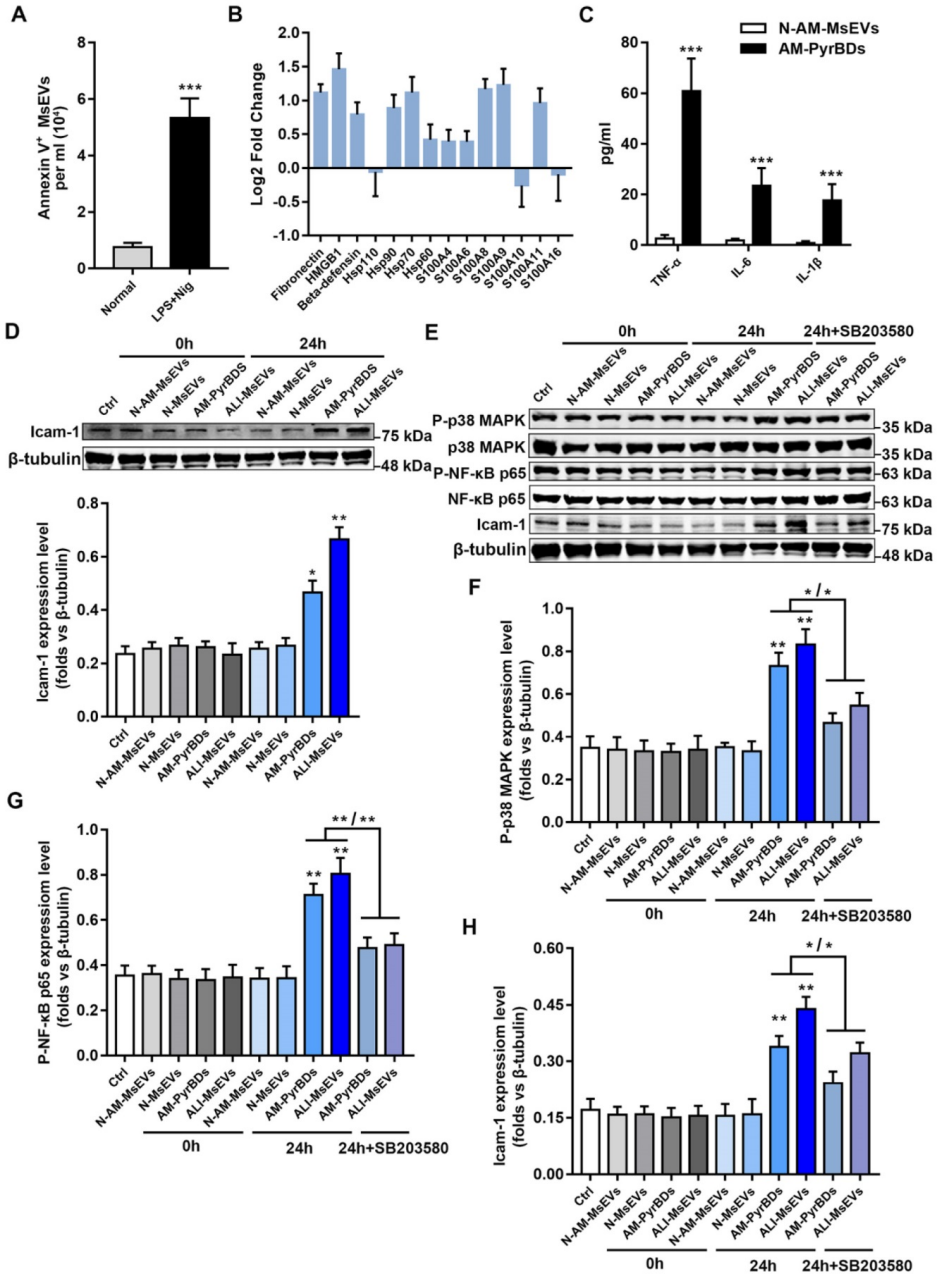
PyrBDs activate the p38 MAPK pathway to promote MLE-12 cell activation. (A) The number of MsEVs was determined by flow cytometry. With the occurrence of pyroptosis, alveolar macrophages derived a large number of MsEVs (PyrBDs). Data are expressed as the mean ± SD, n = 3. (B) PRM for whole-protein analysis of PyrBDs, from which DAMP-related molecular proteins were screened. The results are expressed as log2 fold change (n = 4). (C) TNF-α, IL-6, and IL-1β content of PyrBDs. Compared with MsEVs derived from normal alveolar macrophages (N-AM-MsEVs), MsEVs derived from pyroptotic alveolar macrophages (AM-PyrBDs) contained high levels of TNF-α, IL-6, and IL-1β. Data are expressed as the mean ± SD, n = 6, ***P < 0.001. (D) Immunoblot analysis for icam-1. AM-PyrBDs and ALI-MsEVs (derived from ALI-1h mice) groups showed the promoted expression of icam-1 in MLE-12 cells. N-MsEVs, isolated from BALF of normal mouse; N-AM-MsEVs, derived from normal alveolar macrophages; (E) Immunoblot analysis of phospho-p38 MAPK, p38 MAPK, phospho-NF-κB p65, NF-κB p65, and icam-1. Quantification of the related protein expression showed significant upregulation of phospho-p38 MAPK (F), phospho-NF-κB p65 (G), and icam-1 (H), which was reversed by the inhibitor SB 203580. Data are expressed as the mean ± SD, n = 3. *P < 0.05; **P < 0.01.

The cell line used in the morphological study of PyrBDs was RAW264.7, which was derived from mouse peritoneal macrophages 20. There is no evidence that alveolar macrophages can also form PyrBDs, which prompted us to extract mouse primary alveolar macrophages to determine the release of PyrBDs. Flow cytometry showed that the purity of alveolar macrophages was >95% (Supplemental Figure 3A). The pyroptosis model was established upon LPS + nigericin treatment. The morphologies of the pyroptosis model were analyzed by light microscopy. As expected, the plasma membrane of pyroptotic macrophages became transparent and displayed swelling, with a body similar in shape to a fried egg (Supplemental Figure 3B), whereas untreated macrophages showed a regular round shape. Furthermore, the expression of proteins associated with pyroptosis was assessed by western blotting. Supplemental Figure 3C showed that the expression of cleaved-caspase-1, GSDMD-N, and IL-1β in pyroptotic macrophages was significantly upregulated upon LPS + nigericin treatment compared with that in untreated macrophages. Taken together, these findings indicated that the pyroptosis model of alveolar macrophages was successfully established. Subsequently, we obtained MsEVs, microvesicles, and exosomes derived from pyroptotic alveolar macrophages. Similar to the previous results, MsEVs had the highest protein content ratio (Supplemental Figure 3D and E). We further assessed the quantity of secreted MsEVs by pyroptotic macrophages. As expected, a remarkable increase in Annexin V-positive MsEV concentration was observed following LPS + nigericin treatment, and it was higher than that in the normal group (Figure 5A). In order to distinguish and understand, in the following description, we refer to PyBDs as MsEVs derived from pyroptotic alveolar macrophages.

Whole-protein analysis results confirmed that PyrBDs contained a large number of DAMPs and inflammatory factors such as TNF-α, IL-6, and IL-1β, which is consistent with the previous results of MsEVs (ALI-1h) (Figure 5B and C). To examine the biological function of PyrBDs, MLE-12 pulmonary epithelial cells were co-cultured with PyrBDs isolated from BALF and pyroptotic alveolar macrophages. The expression of intercellular adhesion molecule 1 (icam-1) that is activated on epithelial cells was upregulated after incubation of MLE-12 with PyrBDs for 24 h compared with the controls (Figure 5D).

The damage and activation of epithelial cells usually involve multiple inflammatory pathways, such as the P38 MAPK pathway and the NF-κB pathway 27. To further investigate the molecular mechanism of PyrBDs activating epithelial cells, we determined that MsEVs (ALI-1h) and PyrBDs promoted the phosphorylation levels of NF-κB p65 and p38 MAPK, both of which are involved in the inflammatory response to damage. SB 203580, an inhibitor of p38 MAPK signaling, partly reversed the phosphorylation levels of NF-κB p65 and p38 MAPK as well as icam-1 expression that were upregulated by MsEVs (ALI-1h) and PyrBDs (Figures 5E-H). These findings collectively revealed that PyrBDs activated epithelial cells at least partially via the p38 MAPK signaling pathway.

**Figure 6:**
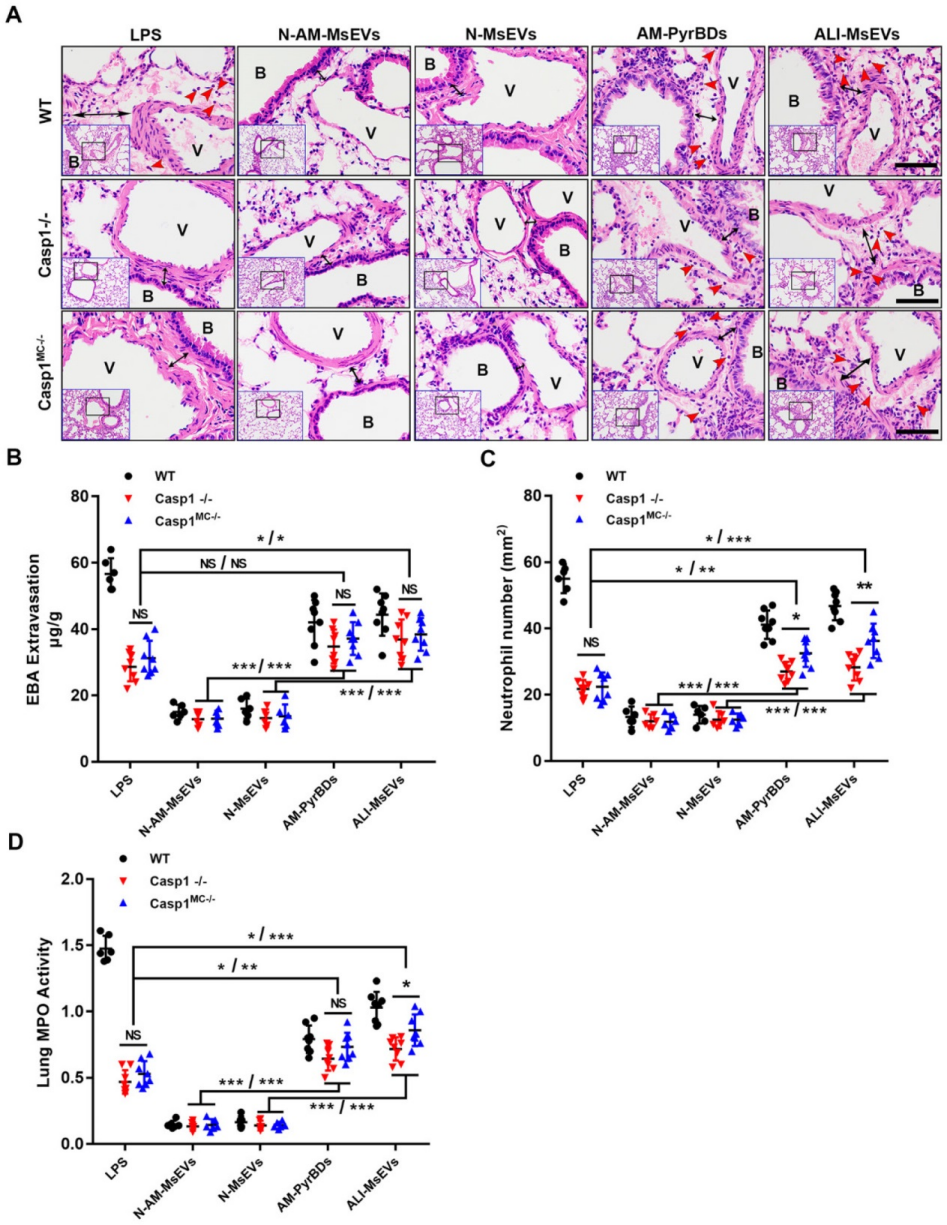
PyrBDs induce vascular leakage and recruit neutrophils. (A) H&E-stained cross-section of the lung from LPS-exposed or PyrBDs treated WT, Casp1^-/-^, and Casp1^MC-/-^ mice at 4 h showed that PyrBDs were able to cause extensive neutrophils infiltration of the lung interstitium. The red arrow points to neutrophils; the black arrow indicates interstitial edema; Bar = 50 μm. B, trachea; V, blood vessel. Quantitative analysis of (B) lung vascular leakage (EBA extravasation), (C) neutrophil infiltration, and (D) lung tissue MPO activity. Compared with the N-MsEV and N-AM-MsEV groups, the PyrBD groups showed more severe vascular leakage and neutrophil infiltration. In addition, PyrBDs reversed the resistance of caspase-1 deficiency to LPS to a certain extent. N-AM-MsEVs, normal alveolar macrophages MsEVs; N-MsEVs, normal mouse MsEVs; AM-PyrBDs, pyroptotic alveolar macrophages MsEVs; ALI-MsEVs, ALI-1h mice MsEVs. Data are expressed as the mean ± SD, NS, no significant difference; *P < 0.05; **P < 0.01; ***P < 0.001.

*PyrBDs induce vascular leakage and recruit neutrophils.* After confirming that PyrBDs could activate inflammatory pathways in vitro to mediate epithelial cell activation, we conducted in vivo experiments and additionally introduced the Casp1^-/-^ and Casp1^MC-/-^ groups. Significant vascular interstitial edema was observed, and free neutrophils were detected in lung tissues by H&E staining after tracheal instillation of AM-PyrBDs and ALI-MsEVs, suggesting that PyrBDs were able to induce vascular interstitial edema and recruit neutrophils. Surprisingly, after 4 h of transmission of PyrBDs in the lung, quantitative analysis of EBA extravasation, the number of neutrophils, and MPO activity revealed that certain pathological damage occurred in the lung tissues of Casp1^-/-^ and Casp1^MC-/-^ mice compared with the LPS group, and Casp1^MC-/-^ mice had more severe damage than Casp1^-/-^ mice, although a few results showed no statistical difference (Figures 6B-D). These results indicated that macrophage-derived PyrBDs might serve as mediators in LPS-induced ALI.

### Characterization of PyBDs

**Figure 7:**
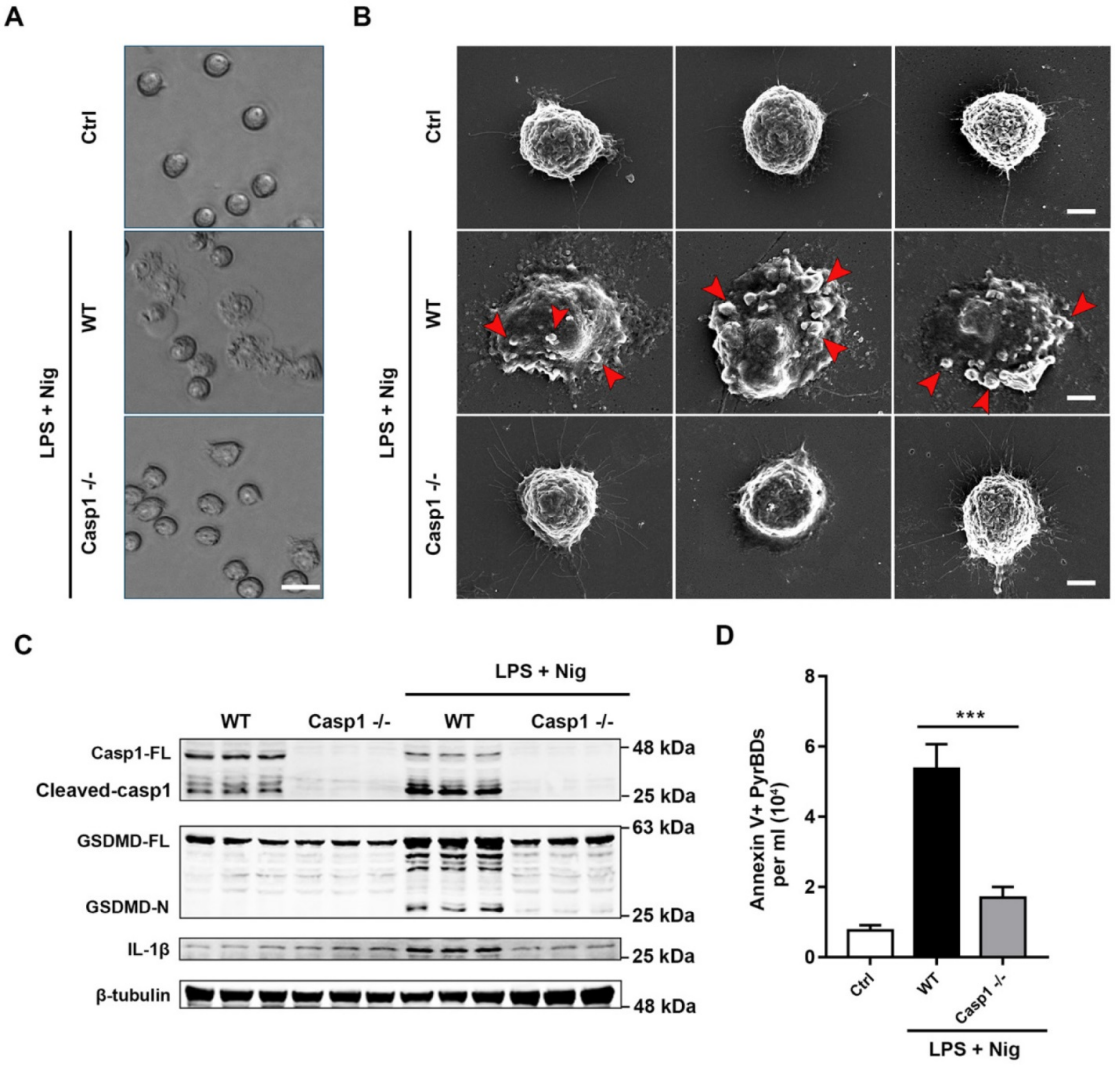
Caspase-1 mediates the formation and release of PyrBDs in macrophages. (A) Observation of the pyroptosis of alveolar macrophages by microscope. (B) Representative scanning electronic microscopy (SEM) images of alveolar macrophages treated with LPS and Nig. Alveolar macrophages from the WT mice showed swelling, release of cytoplasmic content, plasma membrane bubbles, and adhesion of micron-sized vesicles. In the Casp1^-/-^ group, no similar morphological changes were observed. Bar = 5 μm. (C) Immunoblot analysis for pyroptosis-related proteins, caspase-1 full length (Casp1-FL), cleaved caspase-1, GSDMD full length (GSDMD-FL), GSDMD-N-terminal (GSDMD-N), and IL-1β. Data are expressed as the mean ± SD. (D) Lack of caspase-1 led to a decrease in PyrBDs released by alveolar macrophages. Data are expressed as the mean ± SD, ***P < 0.001.

**Figure 8:**
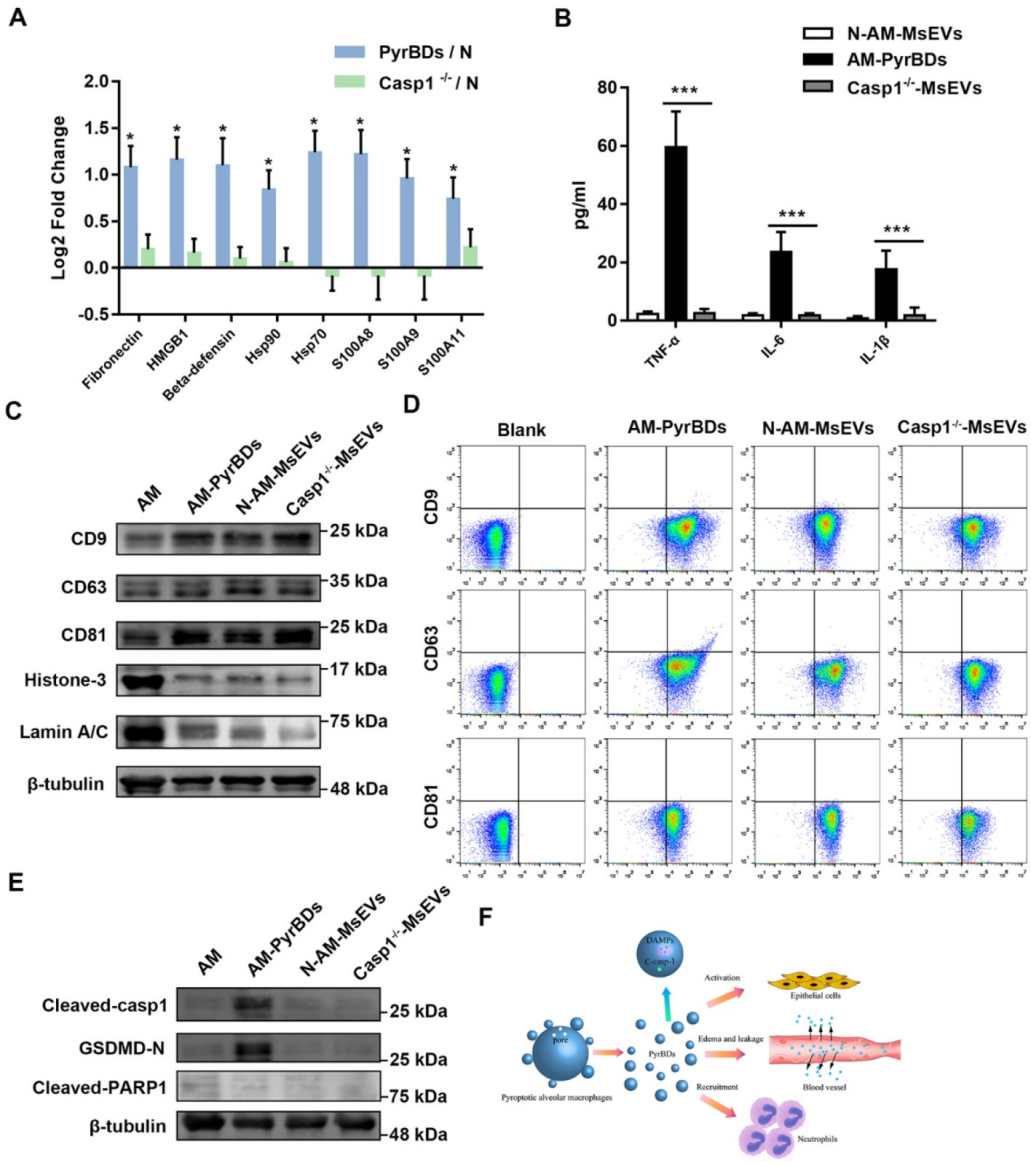
PyrBDs distinctly express cleaved caspase-1 and its substrate proteins. (A) PRM for whole protein analysis of PyrBDs, from which DAMP-related molecular proteins were screened. Loss of caspase-1 led to a decrease in DAMP content. The results are expressed as log2 fold change (n = 4), *P < 0.05. (B) TNF-α, IL-6, and IL-1β content of PyrBDs. Loss of caspase-1 led to decreased expression of inflammatory factors. Data are expressed as the mean ± SD, n = 6, ***P < 0.001. (C) Immunoblot analysis for CD9, CD63, CD81, Histone 3, and Lamin A/C in PyrBDs. (D) Flow cytometry detected the expression of transmembrane proteins CD9, CD63, and CD81 in PyrBDs. (E) Immunoblot analysis for cleaved caspase-1, GSDMD-N, and cleaved PARP1 in PyrBDs. (F) Schematic diagram demonstrating that PyrBDs derived from pyroptotic alveolar macrophages specifically express cleaved caspase-1 and carry DAMPs, mediate epithelial cells activation, increase vascular permeability, and recruit neutrophils.

For further characterization of PyrBDs, we used scanning electron microscopy (SEM) to observe the EV release characteristics of pyroptotic alveolar macrophages. As shown in Figures 7A and B, pyroptotic alveolar macrophages of the WT group displayed swelling and produced multiple bubble-like protrusions, which progressed into micron-sized vesicles encapsulated with cytoplasm contents. In contrast, alveolar macrophages of the Casp1^-/-^ group did not have pyroptotic morphological features. Similarly, the expression levels of cleaved caspase-1, GSDMD-N, and IL-1β in pyroptotic macrophages of the WT group was significantly upregulated upon LPS + nigericin treatment compared with those in the Casp1^-/-^ group (Figure 7C). Quantitative analysis revealed that the concentration of PyrBDs in the WT group was much higher than that in the Casp1^-/-^ group upon LPS + nigericin treatment (Figure 7D), which was consistent with the observation by SEM in Figure 7B. The lack of caspase-1 also led to the reduction of DAMPs in PyrBDs (Figure 8A and B). These results clearly proved that caspase-1 mediated the secretion of PyrBDs by alveolar macrophages.

EVs derived from cell membranes, due to the eversion of phosphatidylserine, release the "eat me" signal. Integrins on the membranes of adjacent cells recognize this signal and mediate phagocytosis 28. Using PKH-67 to label EVs, we observed that in the 2h group, in both EVs derived from alveolar macrophages and PyrBDs-rich samples from ALI mice, MLE-12 cells showed phagocytosis; the 4h group clearly showed micron-sized vesicle signal body in the cytoplasm. The addition of cilengitide (a broad-spectrum integrin inhibitor) significantly inhibited the phagocytosis of PyrBDs by MLE-12 cells (Supplemental Figure 4A), indicating the role of integrin in epithelial cells' recognition of the “eat me” signal released by PyrBDs.

EV markers are crucial for the classification of EVs. Conventional transmembrane proteins, such as CD9, CD63, and CD81, were detected in isolated MsEVs derived from pyroptotic alveolar macrophages (Figure 8C) and from BALF of ALI mice (Supplemental Figure 4B). The results of flow cytometry confirmed such observations (Figure 8D and Supplemental Figure 4C). Hence, regardless of the sample richness in PyrBDs, conventional transmembrane proteins could not be a marker protein of PyrBDs. In addition, we also found that a small amount of nucleoproteins, such as Histone 3 and Lamin A/C, was allocated to these MsEVs (Figure 8C and Supplemental Figure 4B). Activation or cleavage of cellular proteins by executioner caspases such as caspase-1 and caspase-11 (caspase-4/5) can facilitate the process of pyroptosis. Our study focused on the formation of PyrBDs mediated by caspase-1; thus, it is logical to assume that certain cleaved caspase-1 and its substrate proteins can be detected in PyrBDs. To test this, we monitored the expression levels of cleaved caspase-1 and GSDMD-N. In addition, we also detected a non-caspase-1 substrate protein, PARP1, which was located in the nucleus. As expected, cleaved caspase1 and GSDMD-N were easily detected in samples rich in PyrBDs. Cleaved-PARP1 was not detected, which may also be caused by the low content of nucleoprotein in these samples (Figure 8E and Supplemental Figure 4B). These findings suggested that monitoring the presence of cleaved caspase-1 or caspase-1-cleaved proteins could provide additional evidence to determine whether PyrBDs are present in the sample.

## Discussion

The understanding of programmed cell death is still under development 29. Multiple modes of programmed cell death with different molecular mechanisms are involved in the complex process of inflammation, which is dependent on the type and intensity of stimulation and cell type 30. It has been confirmed that pyroptosis is related to acute inflammation and promotes the production and secretion of inflammatory cytokines such as IL-1β and IL-18 12. Interestingly, pyroptotic cells produce PyrBDs that are similar in size to apoptotic bodies; however, research on PyrBDs is limited. This study was the first to characterize and assess the biological activities of PyrBDs in ALI. PyrBD formation was dependent on caspase-1-mediated pyroptosis, and it mainly took place in alveolar macrophages in the early phase of ALI. These micron-sized EVs were shown to be able to promote the activation of epithelial cells via the p38 MAPK signaling pathway, induce vascular interstitial edema, and recruit neutrophils (Figure 8F).

Most studies in the field of EVs have systematically removed micron-sized vesicles from the analysis rather than performing detailed characterization. Owing to their larger size, the existence of PyrBDs is intuitive and visible. Plasma membrane leakage is followed by cytoplasm-flattened alveolar macrophages, production of multiple bubble-like protrusions, and progression to micron-sized PyrBDs upon LPS + Nig challenge. We isolated murine primary alveolar macrophages, which may more accurately reflect changes in macrophages in ALI. Lack of caspase-1 significantly downregulated the quantity of PyrBDs, indicating that the formation of PyrBDs relied on caspase-1. The mechanisms involved in the uncontrolled inflammatory response of ALI include activation of DAMPs and immune cells such as neutrophils. DAMPs, which originate from immune cells that are activated from injured and dying tissues, initiate inflammation and perpetuate the inflammatory response; they also play a critical role in modulating the lung injury response 31. EVs carrying DAMPs, which were released from stressed or injured tissues, were shown to play a substantial role in the induction and persistence of inflammation 32. We found that PyrBDs encapsulated various DAMPs; they also induced vascular interstitial edema and recruited neutrophils, thereby playing a critical role in pulmonary inflammation. PyrBDs can be internalized by epithelial cells both *in vitro* and *in vivo*. Thus, PyrBDs may trigger inflammatory responses through self-breaking or transferring DAMPs to recipient cells. In addition, we described another molecular mechanism which endowed Casp1^-/-^ and Casp1^MC-/-^ mice with strong resistance to LPS, whereas PyrBDs alone were capable of inducing pathological features similar to ALI. This indicated that LPS-induced ALI was probably dependent on pyroptosis of alveolar macrophages in the early phase. The lack of caspase-1 followed by significantly decreased levels of inflammatory cytokines also revealed that alveolar macrophages were the main contributors to inflammatory cytokines in the early phase. Macrophages play a central role in the initiation and resolution of pulmonary inflammation 33, 34. Activated alveolar macrophages play a pro-inflammatory function, together with other cells in the lungs, recruit neutrophils by releasing proinflammatory cytokines, chemokines, and other factors that could affect neutrophil migration^35^. Pyroptotic macrophages will also enhance lung inflammation^36^. The communication between macrophages and other cells in the lung is still in the focus of current studies^8^, ^37^. Our work offers a novel explanation that alveolar macrophages promote cellular crosstalk through the delivery of DAMPs that are mediated by PyrBDs, leading to enhancing pulmonary inflammation.

Major EV subpopulations are recognized as key regulators of intercellular communication. Furthermore, a number of studies have proposed the use of EV subtypes and EV-associated contents as potential biomarkers of pathologic conditions, including cardiovascular, autoimmune, and neurodegenerative diseases^38^-^40^. However, EVs that depend on pyroptosis may have a wide size range because exosomes and microvesicles released during pyroptosis have been detected in some pyroptosis models^17^-^19^. PyrBDs may be one of the largest types of PyrEVs, but many properties of PyrBDs have yet to be fully characterized. It is unclear whether smaller EVs are produced by membrane blebbing during pyroptosis. Some studies have indicated that membrane blebbing is mediated by actin-myosin interactions 41, 42. In addition, the transport of phosphatidylserine outside the membrane is a common feature of apoptotic body and microvesicle formation 43, 44. A similar reverse-budding may occur during exosome formation 45. Transmembrane proteins are not sufficient as distinct markers of PyrBDs because the size of PyrBDs is very similar to that of apoptotic bodies. Hence, if transmembrane protein (Annexin V, CD9, CD63, or CD81)-positive MsEVs are isolated or detected from animals or human samples, it is not feasible to reflect the extent of pyroptosis only by quantification of MsEVs or total protein expression levels. In these cases, as found in our study, in addition to encapsulating a large amount of DAMPs, PyrBDs distinctly express cleaved caspase-1 and its substrate proteins. Therefore, the evaluation of the expression levels of cleaved caspase-1 and its substrate protein or DAMPs suggested that these samples may contain EVs that are produced by pyroptotic cells.

The mechanism by which caspase-1 mediates the formation of PyrBDs remains unclear. Recent studies have shown that in the presence of functional caspase-1, inflammasome activation causes the extracellular appearance of actin that decorates the surface of extending filopodia; actin-associated proteins, including Annexins 1 and 5, are also released and dependent on caspase-1 activity 46, 47. Pyroptosis involves multiple caspase proteins. For instance, GSDMD N-terminal is released after the cleavage by caspase-1 or caspase-11 domain and mediated pyroptosis^12^, ^13^. Hence, it is necessary to determine whether caspase-1 or caspase-11 plays an overlapping role in the formation of PyrBDs derived from pyroptotic alveolar macrophages. GSDMD has been identified as an essential factor downstream of caspase-1 or caspase-11 that mediates pyroptosis and releases interleukin (IL)-1β to the extracellular space. The absence of GSDMD can also prevent the occurrence of pyroptosis 12. Considering the non-selectivity of GSDMD-formed pores, there should not be a substantial increase in intracellular osmolarity 20, suggesting that GSDMD may promote the formation of PyrBDs rather than directly induce the formation of PyrBDs. Although some MsEVs were derived from other cells in ALI, it was not proven that these originated from pyroptotic cells; thus, setting the size limit of PyrBDs to 1-5 μm in diameter for all cell types may not be appropriate. The evaluation system of PyrBDs and other PyrEVs subtypes should be based on the conditions of pyroptosis rather than on making the assumption that PyrBDs are strictly 1-5 μm; however, this warrants further investigation.

In conclusion, our study for the first time revealed the release characteristics and biological function of PyrBDs that originated from alveolar macrophages in the early phase of ALI and further complemented the role of EVs in cellular crosstalk in the process of pulmonary inflammation and injury. In this respect, understanding temporal and spatial dynamics of PyrBDs may provide mechanistic explanations for inflammation spread. PyrBDs have significant effects on other types of cells, particularly on immune cells. Therefore, the role of PyrBDs in inducing innate and adaptive immunity should be further explored.

## Materials and Methods

### Mice

Homozygous Casp1^-/-^ mice were purchased from the Shanghai Model Organism Center, Inc. Casp1^fl/fl^ mice and Lyz2-cre mice were purchased from GemPharmatech Co., Ltd. Macrophage-specific caspase-1-knockout mice (Casp1^MC-/-^) were generated by backcrossing Casp1^fl/fl^ mice and Lyz2-cre mice. C57BL/6J mice were obtained from the Experimental Animal Center of Xuzhou Medical College. All the mice used in this study were 8-12 weeks old and weighed 24-30 g. All experiments were performed in adherence with the National Institutes of Health (NIH Publication, 8th Edition, 2011) guidelines for the use of laboratory animals. The experimental protocols were approved by the Animal Care and Use Committee of Xuzhou Medical University (Ethics Number: 20190615018).

### Reagents and antibodies

LPS (O111:B4), Cilengitide, PKH67 (MINI67) cell linker kit and MPO colorimetric activity assay kit were purchased from Sigma (St. Louis, MO, USA). Nigericin (Nig), liquid calibration beads (1 μm) and counting beads (10 μm) were purchased from Invitrogen (Carlsbad, CA, USA). Liquid calibration beads (5 μm) were purchased from Beijing Haianhongmeng Reference Material Technology Co., Ltd. (Beijing, China). Tissue or cell total protein extraction kit was purchased from Sangon Biotech (Shanghai, China). TNF-α, IL-1β, and IL-6 ELISA kits were purchased from Shanghai Renjie Biotechnology Co., Ltd. (Shanghai, China). Anti-rabbit caspase-1, anti-rabbit IL-1β, anti-rabbit Icam-1, anti-rabbit Histone-H3, anti-rabbit Lamin A/C and anti-mouse β-tubulin antibodies were purchased from Proteintech (Wuhan, Hubei, China). Anti-rabbit p38 MAPK, anti-rabbit phospho-p38 MAPK, anti-rabbit NF-κB p65, and anti-rabbit phospho-NF-κB p65 antibodies were purchased from Cell Signaling Technology (Danvers, MA, USA). Anti-rabbit GSDMD, anti-mouse prosurfactant protein C (SPC) antibodies, and p38 MAPK inhibitor (SB 203580) were purchased from Abcam (Cambridge, MA, USA). Anti-rabbit cleaved-PARP1, anti-rabbit CD9, anti-rabbit CD63, and anti-rabbit CD81 were purchased from ABclonal (Wuhan, Hubei, China). FITC-Annexin V antibody and DAPI staining solution were purchased from KeyGEN Biotech (Nanjing, Jiangsu, China). FITC-anti-mouse F4/80, APC-anti-mouse CD11c, FITC-anti-mouse CD326 (EpCAM), FITC-anti-mouse CD11b, APC-anti-mouse Ly6G, FITC-anti-mouse CD31, FITC-anti-mouse CD9, FITC-anti-mouse CD63 and FITC-anti-mouse CD81 antibodies were purchased from BioLgend (San Diego, CA, USA).

### Mouse ALI model and BALF collection

Mice were anesthetized by intraperitoneal injection of ketamine (100 mg/kg) and xylazine (10 mg/kg) and intubated with a 20G intravenous indwelling needle. LPS (1 mg/kg) was instilled intratracheally. After 1, 2, 4 h, the mice were euthanized by cervical dislocation, and the chest cavity was opened with a midline incision. BALF was performed from the lungs using an intravenous indwelling needle with 1 mL of cold PBS (pH 7.4).

### MsEVs identification and calculation by flow cytometry

EVs were isolated using a sequential centrifugation followed by a sequential filtering. Briefly, BALF samples were centrifuged at 300g for 10 min to remove cell debris, and the supernatant was centrifuged at 2,000g for 20 min to pellet the MsEVs. To isolate microvesicles, the MsEV-depleted supernatant was passed through a 1.2 μm pore filter, then subjected to centrifugation at 12,000g for 40 min. Finally, the resulting supernatant was passed through a 0.2 μm pore filter and ultracentrifuged at 100,000g for 70 min to pellet exosomes. Each type of vesicle was washed with cold PBS. The MsEVs pellets were washed with PBS and then centrifuged twice to remove soluble factors or impurities outside the vesicles. BCA protein concentration determination kit was used to determine the protein concentration of each sample.

The MsEVs pellets were resuspended in 500 μL of PBS, incubated with fluorescence-conjugated antibodies, mixed with 50 µL of counting beads, 50 µL of 1-μm, and 50 µL of 5-μm calibration beads and then analyzed by flow cytometry. MsEVs were defined as Annexin-V-positive and within the size range of 1-5 μm. MsEVs with sizes between 1 and 5 μm were then gated by calibration beads. The number of MsEVs was calculated by the number of counting beads (in line with the manufacturer's instructions) multiplied by the ratio of MsEVs events to counting bead events in flow cytometry plots. The following criteria were used for identification of various cell types: F4/80 and CD11c positivity for alveolar macrophage-derived MsEVs; CD11c positivity and F4/80 negativity for dendritic cells; CD11c negativity and F4/80 positivity for interstitial macrophages; EpCAM positivity for epithelial cells; CD31 positivity for endothelial cells; and CD11b and Ly6G positivity for neutrophils.

### Histologic assessment of lung injury

Left lungs were intratracheally instilled with 4% paraformaldehyde from a 20-cm height, immersed in 4% paraformaldehyde for 24 h, dehydrated, embedded in paraffin, and then the tissues were cut into 4-μm-thick sections and stained with hematoxylin and eosin. Interstitial edema and neutrophil infiltration were used as indices of lung leak and inflammation and evaluated using an optical microscope. Fifteen to 20 images were captured (400× magnification), and quantification of the number of neutrophils was performed by two blinded investigators.

### Evans blue-albumin (EBA) pulmonary transvascular flux measurement

To assess vessel endothelial permeability, we performed the EBA extravasation assay, which is a widely used and well-established in vivo method for quantitative assessment of vascular permeability levels. Briefly, evans blue was injected into anesthetized mouse at a dose of 8 µL/g body weight and allowed to circulate in the blood vessels for 30 min. Intravascular evans blue was washed by PBS perfusion from the right ventricle for 2 min. Mice lungs were excised, weighed, homogenized in 1 mL of PBS, and extracted overnight in 2 mL of formamide at 60°C. Evans blue-bound serum albumin concentration in lung homogenate supernatants was quantified by spectrophotometry at an absorbance wavelength of 620 nm. The concentration of extravasated EBA (microgram of EBA per gram lung) in lung homogenates was calculated against a standard curve.

### MPO assay

Lungs were perfused with PBS to remove all blood, then weighed and stored at -80°C before MPO assay was conducted. MPO activity was detected according to the instructions of MPO colorimetric activity assay kit.

### Quantification of cytokines

TNF-α, IL-1β, and IL-6 levels were measured using quantitative ELISA kits according to the manufacturer's protocols. The supernatant of BALF was collected by centrifugation, and the PyrBDs pellets were resuspended in 100 µL of PBS, disrupted by ultrasonication, centrifuged at 14,000g to obtain the supernatant to detect BALF or to determine PyrBDs internal cytokine content.

### Isolation and identification of primary mouse alveolar macrophages

The mice were sacrificed by cervical dislocation, fixed, the neck skin sliced, and the trachea isolated. An intravenous indwelling needle was inserted into the trachea, and then PBS buffer was used to lavage the alveoli for 8-10 times to collect BALF. The BALF samples were centrifuged at 300g for 10 min, and the red blood cell lysate was added into the resulting precipitate to lyse the red blood cells. The mixture was centrifuged at 300g for 10 min, and then the supernatant was discarded. Then, the cell pellet was resuspended in RPMI-1640 culture medium. Flow cytometry was used to determine the purity of the mouse alveolar macrophages.

### Cell culture and treatment

Mouse pulmonary epithelial cells (MLE-12) were obtained from the Shanghai Zhong Qiao Xin Zhou Biotechnology Co., Ltd (Shanghai, China). Primary alveolar macrophages were harvested from BALF. The cells were cultured in DMEM/F12 and RPMI-1640 culture medium containing 10% fetal bovine serum and incubated in a humidified atmosphere containing 5% CO2. Adding LPS and nigericin induced pyroptosis of alveolar macrophages. Briefly, the cells were primed for 4 h with 1 μg/mL of LPS, and then treated with 10 µM nigericin for 30 min. The samples of the medium were centrifuged (300g, 10 min, 4°C) to remove cells and larger particles, and the resulting supernatant was centrifuged again at high speed (2,000g, 20 min, 4°C) to isolate PyrBDs. PyrBDs were added to the MLE-12 cell culture medium to induce activation or damage (PyrBD : cell ratio was 5 : 1). At the corresponding time point, total protein was extracted.

### Western blot analysis

Proteins from MLE-12 cells, mouse primary alveolar macrophages, and PyrBDs were extracted by using a tissue or cell total protein extraction kit. A BCA protein concentration determination kit was used to determine the protein concentration of each sample. Proteins were separated by SDS-PAGE and transferred onto nitrocellulose membranes. After blocking in 5% nonfat milk for 2 h, the membranes were incubated with primary antibodies against caspase-1, cleaved-PARP1, GSDMD, ICAM-1, IL-1β, NF-κB p65, phospho-NF-κB p65, p38 MAPK, phospho-p38 MAPK, or β-tubulin overnight at 4°C. After washing, the membranes were incubated with fluorescently labeled anti-mouse or anti-rabbit secondary antibodies at room temperature for 1-2 h, and the blot was then imaged using the Odyssey infrared imaging system (Li-Cor). Densitometric analysis of the bands was performed using ImageJ software. Protein levels were calculated from the ratio of corresponding protein/β-tubulin.

### Parallel reaction monitoring (PRM)

Peptides were separated by nano-UPLC liquid phase system EASY-nLC1200 and detected by an online mass spectrometer (Q-Exactive). The analysis used a 100 μm ID × 15-cm reversed-phase column (Reprosil-Pur 120 C18-AQ, 1.9 μm, Dr. Math). The mobile phase A liquid was 0.1% formic acid aqueous solution; B liquid was 0.1% formic acid acetonitrile aqueous solution (acetonitrile, 80%). The column was equilibrated with 100% A liquid. The samples were directly loaded onto the chromatographic column by an autosampler, and then separated by the chromatographic column. The flow rate was 300 nL/min, and the gradient duration was 120 min. The following conditions were used: mobile phase B: 6%-28% for 92 min, 28%-40% for 20 min, 40%-100% for 2 min, 100% for 2 min, 100%-2% for 2 min, and 2% for 2 min. Mass spectrum analysis time was 120 min/sample in positive-ion detection mode. The PRM acquisition method was as follows: MS2 had a resolution of 17,500 at m/z @ 200; AGC was 1E+5; and the maximum ion injection time (Max IT) was 200 ms. The normalized collision energy (NCE) was 27%, and the isolation window was 2.0 m/z. The collected PRM data were imported into Skyline for transition extraction. The transition parameter settings were as follows: parent ion set to +2 and +3 valence; fragment ions set to +1, +2 valence; and selected precursor-last b and y ions for chromatographic peak extraction and quantification. Then, according to the peak shape, intensity, and signal-to-noise ratio of XIC, the final quantitative transition was manually screened. Method match tolerance was 0.005 m/z; MS2 had a resolution of 17,500 at m/z @ 200. Quantitative comparison between different samples was first standardized using internal standard peptides, and then a t test was performed to screen for differential proteins.

### Immunofluorescence staining

To detect the engulfment of PyrBDs in mouse bronchial epithelial cells, PKH67-labeled PyrBDs were infused into the medium (PyrBDs : cell ratio was 10 : 1). The samples were treated with or without cilengitide (10 μM); then, 0, 1, 2, and 4 h post-infusion, the cells were fixed in 4% paraformaldehyde for 15 min. Nuclei were stained with DAPI. After a final washing, the cells were observed under a fluorescence microscope (Olympus).

To detect the engulfment of PyrBDs in the lungs of the mice, PKH67-labeled PyrBDs were infused into mice intravenously through tracheal intubation (10^6^ PyrBDs per mouse). At 4 h post-infusion the lungs were fixed in 4% paraformaldehyde, and then frozen sections were prepared. The sections were fixed with 4% paraformaldehyde for 15 min, permeabilized with Triton X-100 (0.1%), and blocked with a solution containing 5% bovine serum before applying the primary antibody. The specimens were incubated with anti-mouse SPC antibody for 12 h at 4°C and incubated with secondary antibodies (Alexa Flour 594 donkey anti-mouse antibody) under light-protected conditions for 1 h at room temperature. Nuclei were stained with DAPI. After a final washing, coverslips were mounted on the slides using 50% glycerin. Then, the sections were observed under a fluorescence microscope (Olympus).

### Scanning electron microscopy (SEM)

The samples were fixed with 2.5% glutaraldehyde overnight and then rinsed with PBS three times. Next, they were dehydrated through an ethanol gradient (30%, 50%, 70%, 95%, and 100%) and dried by tertiary butanol method. Dried specimens were sputter-coated with gold-palladium and imaged with an SEM system (SEM-Teneo VS).

### Transmission electron microscopy (TEM)

A drop of the prepared suspension containing PyrBDs was deposited onto a copper mesh coated with a formvar film for 5 min, which was then placed in a Petri dish with filter paper and then air-dried. Afterward, the sample was placed into the sample tank of the TEM (Tecnai G2 Spirit Twin) and examined.

### Statistical analysis

Data were expressed as mean ± standard deviation (SD). Multiple group comparisons were evaluated using one-way ANOVA followed by least-significant difference t test for post-hoc analysis. Data between two independent groups were compared using a two-tailed Student's t test. Analyses were performed using SPSS 25 software (Chicago, IL, USA). Differences with *P* < 0.05 were considered statistically significant.

## Supplementary Material

Supplementary figures.
