# Supplementary material for: Caspase-1-mediated extracellular vesicles derived from pyroptotic alveolar macrophages promote inflammation in acute lung injury
Source: Int J Biol Sci. 2022 Jan 24;18(4):1521–38. doi: 10.7150/ijbs.66477 (PMC8898368; doi:10.7150/ijbs.66477)
Supplement: Supplementary file 1 — Supplementary figures. [file ijbsv18p1521s1.pdf]

Supplemental Figure 1

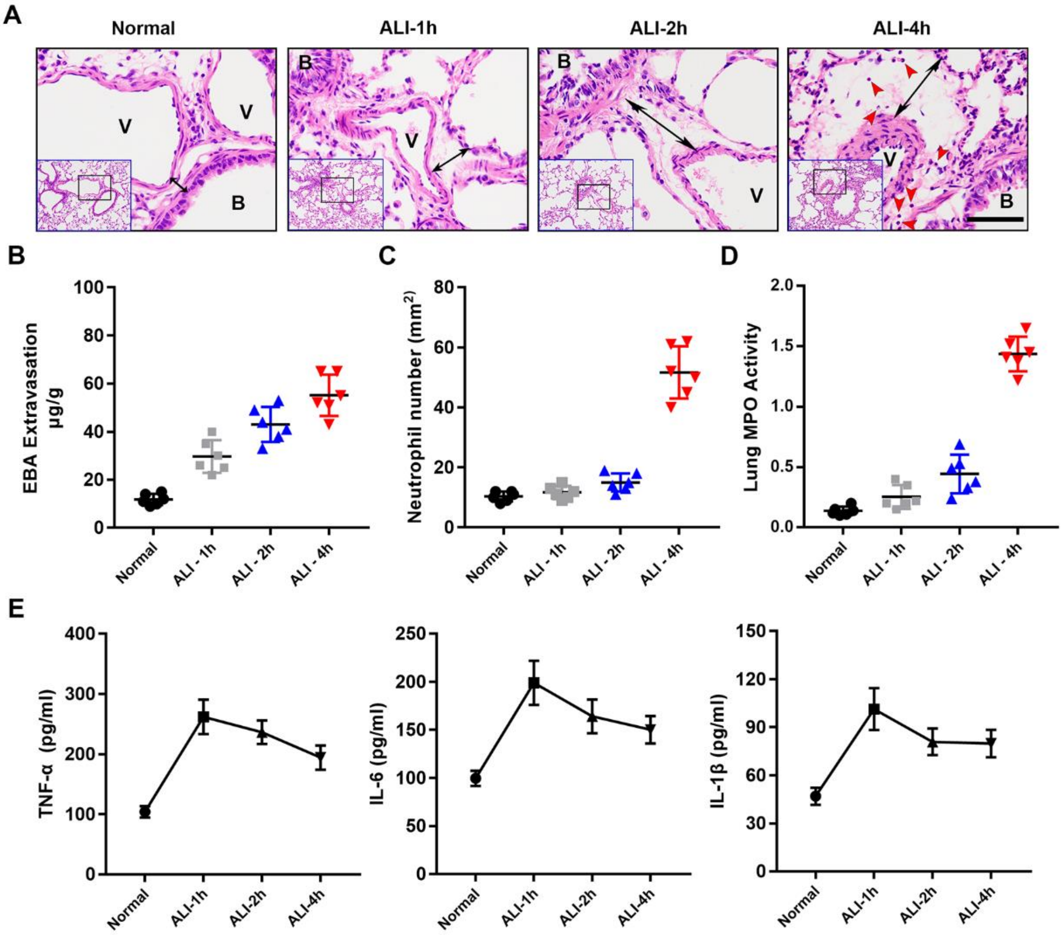

Supplemental Figure 1: Pathological characteristics of LPS-induced acute lung injury in mice. (**A**) H&E-stained cross-sections of the lung from control mice and LPS-exposed mice at 1, 2, and 4 h show interstitial edema (indicated by black arrows) in the ALI groups. The red arrow points to neutrophils. Scale bars: 50 μm. B, trachea; V, blood vessel. Images are representative of six animals. (**B**) Assessment of the degree of lung vascular leakage (EBA extravasation). (**C**) Quantitative analysis of neutrophils infiltration of the lungs. (**D**) Quantitative analysis of lung tissue MPO activity. (**E**) Quantification of cytokines (n=6). TNF-α, IL-1β, and IL-6 expression levels increased in the ALI groups, and the highest expression was observed in the ALI-1h group. Data are expressed as the mean ± SD.

Supplemental Figure 2

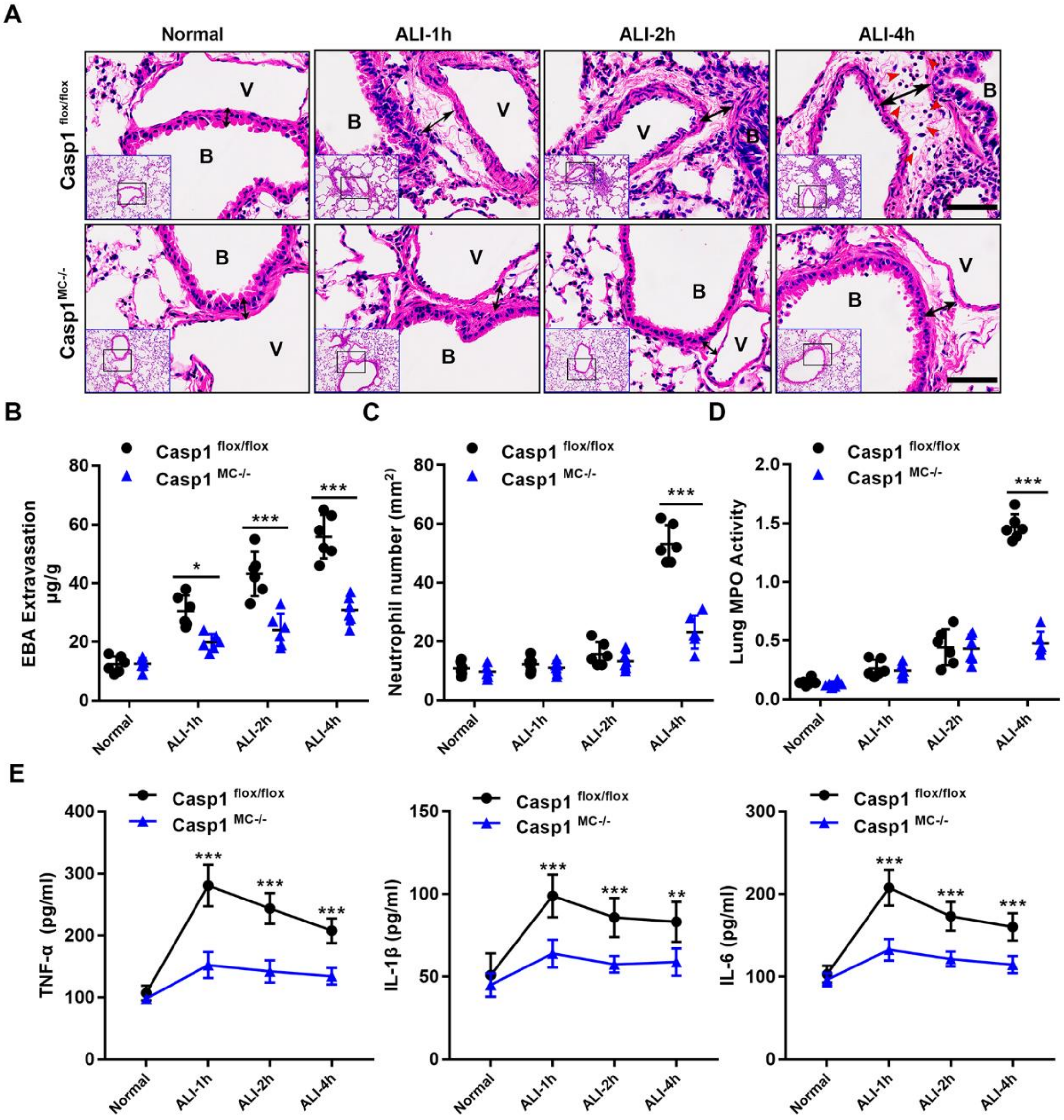

Supplemental Figure 2: Effect of LPS on the lung tissue of mice lacking caspase-1 in macrophages. (**A**) H&E-stained cross-section of the lung from LPS-exposed Casp1$^{flox/flox}$ and Casp1$^{MC-/-}$ mice at 1, 2, and 4 h shows interstitial edema (indicated by black arrows) in the ALI groups. The red arrow points to neutrophils. Scale bars: 50 μm. B, trachea; V, blood vessel. Images are representative of six animals. Quantitative analysis of (**B**) lung vascular leakage (EBA extravasation), (**C**) neutrophil infiltration, and (**D**) lung tissue MPO activity. Lack of caspase-1 in macrophages significantly reduced LPS-induced vascular leakage and neutrophil infiltration. (**E**) Quantification of cytokines. TNF-α, IL-1β, and IL-6 expression increased in the Casp1$^{flox/flox}$ groups compared with the Casp1$^{MC-/-}$ groups (n=6). Data are expressed as the mean ± SD. $^{**}P < 0.01$; $^{***}P < 0.001$.

Supplemental Figure 3

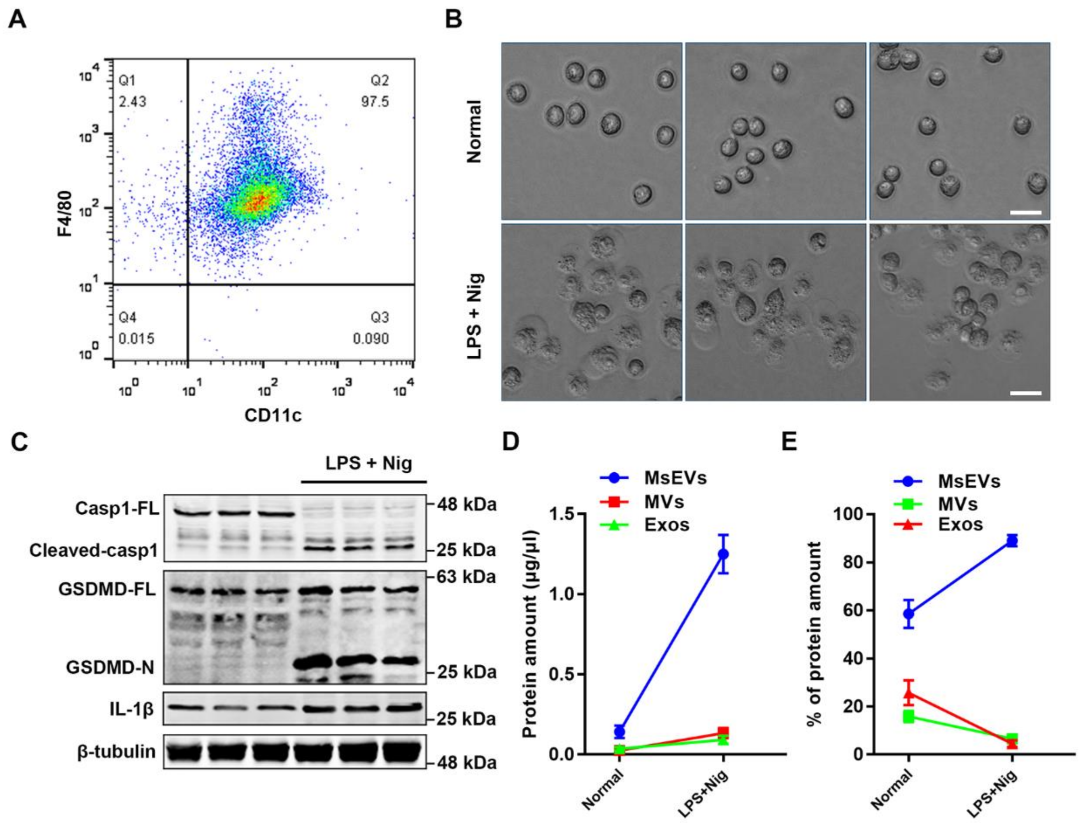

Supplemental Figure 3: Primary alveolar macrophages release large amounts of MsEVs during pyroptosis in vitro. (**A**) Identification of primary alveolar macrophages using flow cytometry. $CD11c^+$ and $F4/80^+$ events stand for primary alveolar macrophages. (**B**) Observation of the pyroptosis of alveolar macrophages by microscopy. The cells were primed with LPS (1 μg/mL) for 4 h and subsequently treated with Nig (5 μM) for 30 min or no treatment. (**C**) Immunoblot analysis for caspase-1 full length (Casp1-FL), cleaved caspase-1, GSDMD full length (GSDMD-FL), GSDMD-N-terminal (GSDMD-N), and IL-1β. The related proteins representing the degree of pyroptosis significantly increased. (**D**) Protein concentration of MsEVs, microvesicles and exosomes. (**E**) The protein concentration percentages of each type of EVs.

Supplemental Figure 4

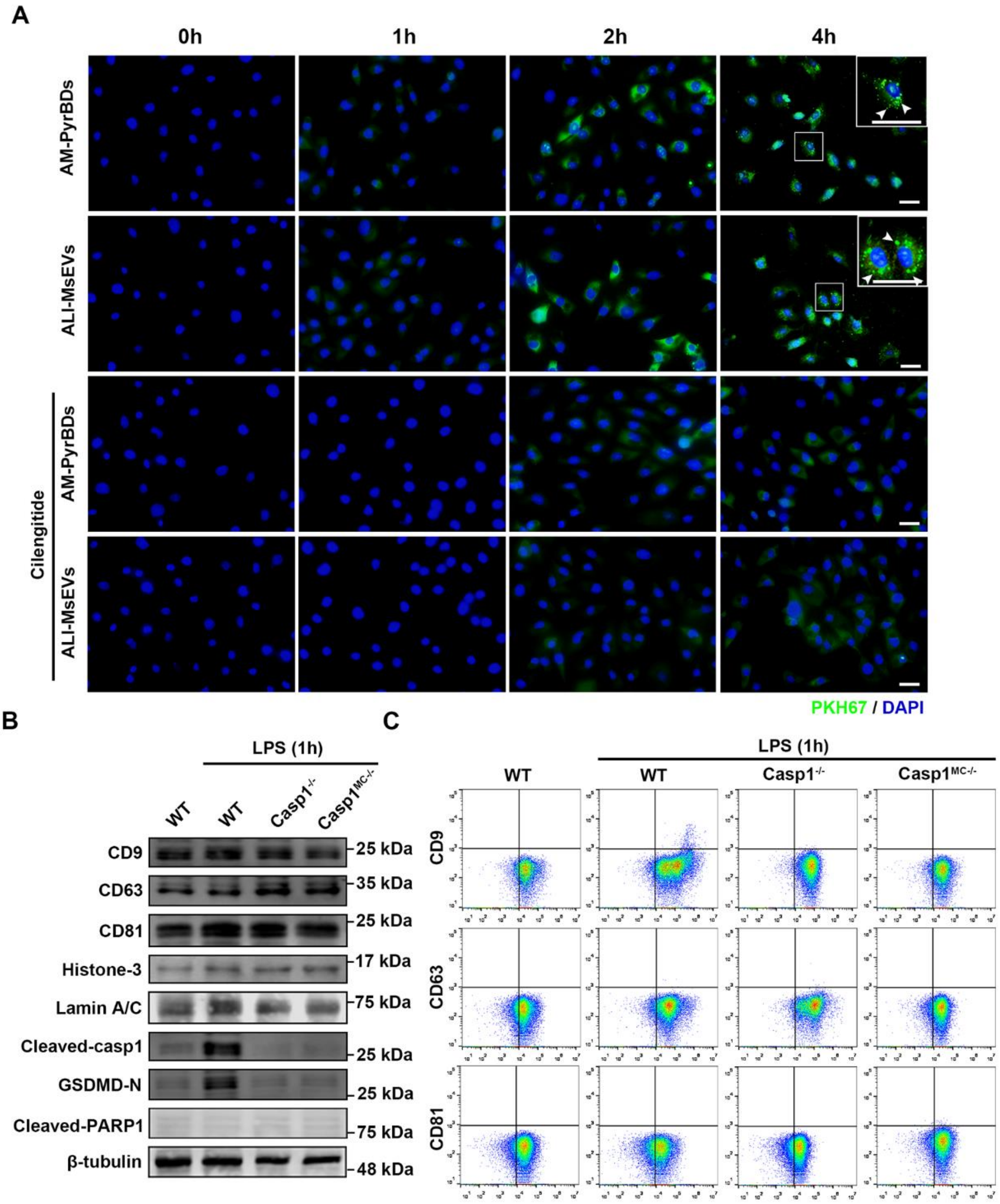

Supplemental Figure 4: Characterization of PyrBDs. (**A**) PKH67-labeled PyrBDs were collected from LPS + Nig-treated alveolar macrophages (AM-PyrBDs) or mice exposed to LPS for 1 h (ALI-MsEVs); immunofluorescence staining showed the localization of PyrBDs in the cytoplasm. Cilengitide significantly suppressed this phenomenon. The white arrow points to undegraded PyrBDs (green); bar = 20 μm; DAPI (blue). (**B**) Immunoblot analysis for CD9, CD63, CD81, Histone 3, Lamin A/C, cleaved caspase-1, GSDMD-N, and cleaved PARP1 in PyrBDs isolated from multiple genotype mice. (**C**) Flow cytometry detected the expression of transmembrane proteins CD9, CD63, and CD81 in PyrBDs isolated from multiple genotype mice.
